# Malignant salivary gland tumors of the tongue: Analysis of 29 cases

**DOI:** 10.4317/medoral.27887

**Published:** 2026-03-07

**Authors:** Kuauhyama Luna-Ortiz, Salvador S Yescas-Castellanos, Zelik Luna-Peteuil, Dorian Y García-Ortega

**Affiliations:** 1 Department of Head and Neck Surgery at Instituto Nacional de Cancerología, México; 2 Department of Surgery (Head and Neck) at the Hospital Manuel Gea Gonzalez, México; 3 Medical School at Universitatea de Medicină și Farmacie “Grigore T. Popa”, Romania; 4 Department of Surgical Oncology at Instituto Nacional de Cancerología, México

## Abstract

**Background:**

Minor salivary gland carcinomas of the tongue are rare tumors with diverse clinical and histological features and poor prognosis. Evidence regarding their characteristics and survival is limited. This study aimed to describe their clinical presentation, histology, and survival.

**Material and Methods:**

We conducted a retrospective study of 29 patients with a histopathological diagnosis of minor salivary gland carcinoma of the tongue, treated at a referral cancer center between January 1990 and December 2024. Clinical, histological, therapeutic, and survival data were evaluated using the Kaplan-Meier method.

**Results:**

The median age was 61 years (range, 21-99), with a female predominance (62.1%). The base of the tongue was the most common site (65.5%). Adenoid cystic carcinoma was the most frequent histology (48.3%), followed by adenocarcinoma (27.6%) and mucoepidermoid carcinoma (24.1%). Most patients were diagnosed at advanced stages (III-IV, 65.4%), with node involvement in 41.4% of cases. Surgery was the primary treatment modality (38%), and radiotherapy was administered in 48.3% of cases. Five-year disease-free survival was 70%, while overall survival was 35%, with a median of 87 months. Moderately differentiated tumors showed a trend toward better survival, without statistical significance.

**Conclusions:**

Minor salivary gland carcinomas of the tongue are rare and frequently diagnosed at advanced stages. Prognosis is largely influenced by histology and tumor differentiation, highlighting the importance of long-term follow-up and multicenter studies to more reliably identify prognostic factors.

## Introduction

Malignant salivary gland tumors account for 3-5% of all head and neck cancers, with up to 20% originating from minor salivary glands (MSG).(1 , 2) Although rare, nearly 80% of MSG tumors are malignant, in stark contrast to major salivary glands, where up to 80% are benign. MSG tumors tend to display considerable heterogeneity in clinical presentation and histology.(3 , 4) MSG are distributed throughout the upper aerodigestive tract, with approximately 450-750 glands in the sinonasal cavity, oropharynx, larynx, and trachea, but most are located in the oral cavity-particularly at the base of the tongue.(5) The base of the tongue is the second most common oral site for MSG tumors after the palate, with adenoid cystic carcinoma as the predominant histology, followed by mucoepidermoid carcinoma.(6 , 7)

Although the epidemiology and prognostic factors of squamous cell carcinoma (SCC) of the tongue are well known, data on MSG carcinomas of the tongue is limited, mostly from case reports or older retrospective studies.(8 , 9) Management generally consists of complete surgical excision, followed by radiotherapy for high-risk histological features.(10 , 11) This study aimed to describe the clinical, demographic, and survival characteristics of MSG tumors of the tongue treated over 34 years.

## Material and Methods

We conducted a retrospective study of medical records from a database of 900 patients with tongue cancer between January 1990 and December 2024, selecting those with a diagnosis of MSG tumors. Data collected included demographic variables, symptoms, diagnostic methods, histopathological characteristics, and initial and adjuvant treatment. This study was reviewed and approved by the Institute's Research and Ethics Committee and was registered under No. 034/20. We included de novo patients at our institution as well as those previously treated elsewhere who presented with persistent or recurrent disease.

Statistical analysis was performed using SPSS® Statistics, version 20 (IBM SPSS, Armonk, NY, USA). Survival was calculated using the Kaplan-Meier method.

## Results

Demographic and Clinical Characteristics

A total of 29 patients with a histopathological diagnosis of MSG carcinoma of the tongue were included. Table 1 summarizes the case distribution by gender, showing a female predominance (62.1%), as well as the main clinical characteristics of the tumors.

Table 1The most common tumor location was the base of the tongue, including the lateral border, observed in 9 cases (31%). In most patients (79.3%), the tumor was confined to the primary site. Computed tomography (CT) was the most frequently used diagnostic modality, performed in 12 patients (41.4%).

Histology

Four main histological types were identified. Adenoid cystic carcinoma was the most common, accounting for 48.3% of cases based on the final report, followed by adenocarcinoma (8 cases, 27.6%) and mucoepidermoid carcinoma (7 cases, 24.1%) (Table 2). Moderately differentiated tumors were the predominant grade, representing 44.8% of cases.

Table 2TNM Staging

Table 3 shows the distribution of patients according to TNM classification and clinical stage, with 65.4% diagnosed at advanced stages (III and IV). Staging could not be determined in 17.2% of cases. Clinical lymph node involvement (N+) was observed in 12 patients (41.4%) with palpable nodes at diagnosis: 5 patients (35.7%) with adenoid cystic carcinoma, 5 (35.7%) with adenocarcinoma and 2 (28.6%) with mucoepidermoid carcinoma.

Table 3Treatment

Surgery was the most common treatment, performed in 11 patients (38%), with partial and hemiglossectomy as the primary procedures, as detailed in Table 3.

Radiotherapy was administered to 14 patients (48.3%), of whom 9 (31%) received it as definitive treatment and 5 (17.2%) as adjuvant therapy.

Disease-Free Survival

**Figure 1:**
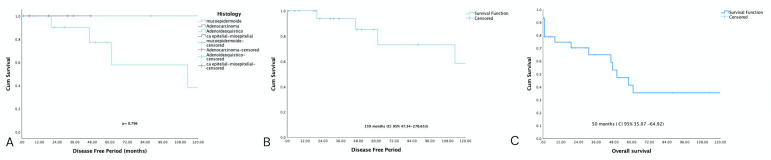
A) Disease-free survival in all patients. B) Disease-free survival by histology. C) Overall survival in patients, according to Kaplan-Meier estimates.

The median time to recurrence for the entire cohort was 35 months (range, 4.3-65.6). When stratified by histology, the median time to recurrence was 19 months (range, 0-47) for mucoepidermoid carcinoma, 5 months (range, 0-10.3) for adenocarcinoma and 50 months (range, 31.5-68.4) for adenoid cystic carcinoma (Figure 1A).

[caption id="attachment_2027" align="alignnone" width="300"]1 Overall survival[/caption]Overall survival (OS) for the entire cohort was 87.39 months (range, 48.47-126.3). By histologic subtype, the median OS was 88.45 months (range, 17.7-159.1) (Figure 1B) for mucoepidermoid carcinoma, 20.87 months (range, 9.88-31.86) for adenocarcinoma, and 108.46 months (range, 49.15-167.76) for adenoid cystic carcinoma (Figure 1C).

## Discussion

Carcinomas originating from MSG of the tongue constitute a heterogeneous group of tumors with variable clinical presentation, but with biological behavior specific to each histological type and anatomical subsite of the primary tumor.(5 , 12)

In this series, the mean age at presentation was 61 years, ranging from 21 to 99 years. This finding is consistent with previous reports, as most authors have described a peak incidence in the sixth decade of life.(2 , 7)

Female patients predominated (62.1%, ratio 1.6:1), which is consistent with the findings of Poissonnet et al. who reported a ratio of approximately 1:1.5.(13) Unlike most head and neck cancers, carcinomas originating from MSG are not associated with tobacco or alcohol use. Identified risk factors include exposure to ionizing radiation, particularly from radiotherapy, as well as during dental or cervicofacial radiological procedures. Occupational exposures have also been proposed as potential risk factors, including cleaning personnel, electrical equipment assemblers, and building painters, among others. In this series, the base of the tongue was the most common anatomical site of origin (65.5%), in line with previous reports,(13) as these tumors typically arise at the base of the tongue, where MSG are numerous. In contrast, MSG are sparse in the mobile tongue; in our series, 34.5% of cases occurred there, compared with 14.6% reported in the literature.(6 , 13)

This series covers a long time period during which diagnosis approaches evolved: Initially, diagnosis was clinical, followed by the introduction of CT imaging and later MRI. Despite these developments, CT remained the imaging modality of choice in this series. Currently, however, we consider both modalities complementary, particularly for tumors located in areas difficult to assess on physical examination and for evaluating tumor depth.(1)

In the literature, adenoid cystic carcinoma remains the most common tumor, consistent with our findings (48.3%), followed by mucoepidermoid carcinoma. In contrast, in this series adenocarcinoma NOS was the second most common subtype (27.6%).(9 , 13) Most patients presented with advanced stages (III and IV) in 65.4% of cases based on T classification, with node involvement (N+) observed in 41.4%. These findings are consistent with previous reports, and may be explained by the tumor location and the nonspecific nature of symptoms.(1 , 13 , 14)

Although the evidence is limited, solely based on retrospective studies, wide surgical resection with negative margins remains the preferred treatment whenever feasible.(1 , 13) In our series, 38% of patients underwent surgery, primarily partial or hemiglossectomy, similar to the 39% reported by Iyer et al.(11) Thus, surgery remains the cornerstone of primary treatment; however, due to the location of these tumors, achieving adequate resection may require complex procedures. Consequently, a considerable proportion of patients received adjuvant and/or definitive radiotherapy (48%), despite the low radiosensitivity and chemosensitivity of these tumors compared with squamous cell carcinoma.(10 , 15) Another therapeutic option, not available at our institution or in many centers, is proton and carbon ion therapy, which has shown a 5-year local control rate of 92%.(16)

Lymph node metastases were observed in 62.5% of adenocarcinoma cases, slightly higher than the 59% reported in the literature. In mucoepidermoid carcinoma, node involvement occurred in 28.6% of patients, compared with 41% in published series, particularly in intermediate-and high-grade tumors, where rates may reach up to 46%. This variability is influenced by tumor site, as lesions of the base of the tongue, palate, and retromolar trigone carry a higher risk of node spread due to their rich lymphatic drainage, even when of intermediate grade. In contrast, adenoid cystic carcinoma typically shows node metastases in approximately 10.9% of cases; however, in our series, the rate was 35.7%.(17 - 20)

Distant metastases, on the other hand, have been rarely reported in adenocarcinoma and mucoepidermoid carcinoma.(19 , 20) In adenoid cystic carcinoma, distant metastases are more common, occurring in up to 52% of patients, most frequently in the lungs (84.2%), which reflects a distinct biological pattern, with distant spread predominating over local recurrence.(17 , 18) These findings underscore the importance of close and prolonged follow-up as local recurrence or distant metastases may develop many years, or even decades, after treatment.(18 , 20)

In this series, no distant metastases were observed in mucoepidermoid carcinoma, compared with 2.2% reported in the literature. Distant metastases occurred in 21.4% of adenoid cystic carcinoma cases, exclusively to the lungs, compared with 52% in previous reports. Adenocarcinoma showed distant spread in 25% of patients, involving the liver and adrenal glands, consistent with the 21% reported by De Luca et al.,(20) with variation depending on histological grade. These findings highlight the distinct biological behavior of each tumor subtype: Adenoid cystic carcinoma shows a predilection for hematogenous spread to the lungs, whereas adenocarcinoma more commonly metastasizes to the liver-a pattern confirmed in our series.

In our series, the five-year disease-free survival was 70%, with a median follow-up of 159 months. Kaplan-Meier analysis by histology revealed no statistically significant differences (p=0.796). Iyer et al. reported a median follow-up of 86 months (range, 12-249), with five-year disease-free survival of 87% and 67%. In their series, 34% of patients experienced recurrence, including 7 local, 6 regional, and 13 distant cases.(11)

The five-year overall survival rate was 35%, with an estimated median of 87 months. Kaplan-Meier estimates revealed subgroup differences, with women and smokers showing longer median survival. In contrast, Iyer et al. did not find significant differences in overall survival or disease-free survival between adenoid cystic carcinoma and mucoepidermoid carcinoma at 5 years; however, at 10 years, patients with adenoid cystic carcinoma exhibited poorer outcomes.(11)

Regarding prognostic factors, it has generally been demonstrated that the degree of histological differentiation plays an important role in overall survival. Hay et al. analyzed various factors and identified only histological differentiation as an independent prognostic factor in multivariate analysis. (16) In our series, unlike the findings reported by Poissonnet et al., (13) histological grade was not significantly associated with clinical outcome (p=0.65). However, a nonsignificant trend toward better survival was observed in moderately differentiated tumors compared with poorly differentiated ones, likely due to the limited sample size.

Similarly, histology, histological grade, and clinical stage did not reach statistical significance, likely due to the limited sample size. In contrast, Lloyd et al. identified male gender, histological subtypes (adenoid cystic carcinoma and mucoepidermoid carcinoma), degree of differentiation, and T stage greater than 2 as significant factors in their multivariate analysis.(22) Moretz-Sohn et al.(14) reported that alcohol use, adenocarcinoma subtype, advanced stages (III or IV), tumor location at the base of the tongue or floor of the mouth, as well as vascular invasion, were significantly associated with an increased risk of node metastasis. In our series, these associations could not be confirmed through multivariate analysis, which constitutes a limitation of the present study.

## Conclusions

Malignant minor salivary gland tumors of the tongue are rare, occurring predominantly in women and most often arising at the base of the tongue. However, our series demonstrated twice as many cases in the mobile tongue (34.5%), with adenoid cystic carcinoma as the most common subtype, followed by adenocarcinoma, differing from other cohorts. Due to their clinical heterogeneity, these tumors generally carry an unfavorable prognosis; however, we observed a trend toward improved survival in patients with moderately differentiated tumors. Given the aggressive biological behavior of these histologies, characterized by both local and distant recurrence, long-term follow-up is essential. Our findings contribute to the clinical and prognostic characterization in Latin American populations and highlight the need for multicenter studies with larger sample sizes to strengthen prognostic evidence.

## Figures and Tables

**Table 1:** Table Clinical and histological characteristics.

Category	Variable n (%)
Gender	
Male	11 (37.9)
Female	18 (62.1)
Side	
Right	15 (51.7)
Left	14 (48.3)
Tumor location	
Base of tongue + lateral border	9 (31)
Lateral border	9 (31)
Base of tongue	5 (17.2)
Base of tongue + floor of mouth	5 (17.2)
Floor of mouth	1 (3.4)
Clinically positive neck	
No	17 (58.6)
Yes	12 (41.4)
Tumor extension	
Confined	23 (79.3)
Jaw	5 (17.2)
Adjacent	1 (3.4)
Diagnostic method	
CT	12 (41.4)
Clinical	10 (34.5)
MRI	6 (20.7)
US	1 (3.4)
Smoking	
No	16 (55.2)
Yes	13 (44.8)
Alcohol use	
No	19 (65.5)
Yes	10 (34.5)
Histology	
Adenoid cystic carcinoma	14 (48.3)
Adenocarcinoma	8 (27.6)
Mucoepidermoid carcinoma	7 (24.1)
Perineural invasion	
Not assessable	13 (44.8)
No	11 (37.9)
Yes	5 (17.2)
Lymphovascular invasion	
Not assessable	13 (44.8)
No	13 (44.8)
Yes	3 (10.3)
Margins	
Not Reported	19 (65.5)
>5mm	5 (17.2)
<5mm >2.7mm	2 (6.9)
<2.7mm	3 (10.3)
Histological grade	
Not assessable	8 (27.6)
G0	1 (3.4)
G1 Well differentiated	2 (6.9)
G2 Moderately differentiated	13 (44.8)
G3 Poorly differentiated	5 (17.2)
Metastasis	
Lymph node metastasis	
Adenoid cystic carcinoma	
Yes	5 (35.7)
No	9 (64.3)
Adenocarcinoma	
Yes	5 (62.5)
No	3 (37.5)
Mucoepidermoid	
Yes	2 (28.6)
No	5 (71.4)
Distant metastasis	
Adenoid cystic carcinoma	
Yes	3 (21.4)
No	11 (78.6)
Adenocarcinoma	
Yes	2 (25)
No	6 (75)
Mucoepidermoid	
Yes	0 (0)
No	7 (100)
Site of metastasis	
Adenoid cystic carcinoma	
Lungs	3 (100)
Adenocarcinoma	
Liver	1 (50)
Adrenal glands	1 (50)

1

**Table 2:** Table TNM Staging.

	NX	N0	N1	N2A	N2C	Total n (%)	II	III	IVA	IVB	IVC	Not assessable	Total n (%)
TX	4	1	0	0	0	5 (17.2)	0	0	0	0	0	5	5 (17.2)
T1	0	1	1	0	1	3 (10.3)	0	1	1	1	0	0	3 (10.3)
T2	0	5	1	0	1	7 (24.1)	5	1	1	0	0	0	7 (24.1)
T3	0	4	1	2	0	7 (24.1)	0	4	2	0	1	0	7 (24.1)
T4A	0	2	2	0	3	7 (24.1)	0	0	7	0	0	0	7 (24.1)
Total	4	13	5	2	5	29 (100)	5	6	11	1	1	5	29 (100)

2

**Table 3:** Table Treatment.

	n (%)
Initial treatment	
Surgery	11 (38)
Watchful waiting	9 (31)
Radiotherapy	5 (17.2)
Concomitant chemoradiotherapy	4 (13.8)
Type of surgery	
Partial glossectomy	3 (10.3)
Hemiglossectomy	3 (10.3)
Wide local resection of tongue + floor of mouth resection	2 (6.9)
Hemiglossectomy + segmental mandibulectomy + floor of mouth resection	2 (6.9)
Total glossectomy	1 (3.4)
Regional control	
None	23 (79.3)
Selective neck dissection	2 (6.9)
Supraomohyoid neck dissection	2 (6.9)
Modified Radical Neck Dissection + Bilateral Supraomohyoid Neck Dissection	2 (6.9)
Radiotherapy	
No	15 (51.7)
Yes	14 (48.3)
Radiotherapy modality	
None	15 (51.7)
Definitive	9 (31)
Adjuvant	5 (17.2)

3

## Data Availability

Declared none.
